# Editorial: Microbiome metabolites in health and disease

**DOI:** 10.3389/fmicb.2023.1270001

**Published:** 2023-09-12

**Authors:** Emanuel E. Canfora, Mark A. Feitelson, Alla Arzumanyan

**Affiliations:** ^1^Human Biology, School for Nutrition and Translational Research in Metabolism, Maastricht University Medical Center+, Maastricht, Netherlands; ^2^Department of Biology, College of Science and Technology, Temple University, Philadelphia, PA, United States

**Keywords:** microbial metabolites, celiac disease, sepsis, gut barrier function, short chain fatty acids, acute liver failure (ALF)

The gut microbiome is a major factor influencing host health and disease. Disturbances in the composition and functionality of the gut microbiota could potentially contribute to disruptions in host metabolism, affecting the functionality of various organs such as the gut, adipose tissue, muscle, liver, kidneys, and brain. Therefore, it is suggested that the gut microbiome is involved in the progression of diseases such as autoimmunity, metabolic syndrome, chronic inflammation, neurodegeneration, allergic reactions, and cancer.

In the past two decades, the role of gut microbiome-derived metabolites in maintaining health and in how alterations in these metabolites promote the development of diseases has been widely studied. However, substantial work remains toward understanding the interaction between microbial-derived metabolites and host metabolism as well as the underlying mechanisms in health and disease.

Thus, the main aim of this Research Topic was to facilitate an understanding of (1) how the various functional characteristics of bacterial metabolites impact organ systems, (2) whether different diseases can be characterized by altered ratios of bacterial populations and their metabolites in clinical samples, and (3) if these alterations could serve as a basis for the development of microbiome metabolite-based therapeutics for a wide range of diseases. Within this topic, seven articles have been published that have expanded our knowledge on the impact of gut microbial metabolites in different disease states, using preclinical models and advanced analytic tools.

Shi et al. analyzed the fecal microbial composition and metabolome characteristics of Chinese patients diagnosed with celiac disease combining data on 16S rDNA sequencing and metabolomics. The authors' goal in the case–control study (30 healthy controls and 30 patients with celiac disease) was to identify potential biomarkers that could be used for the diagnosis of celiac disease. First, the study revealed differences in gut microbiota composition between the celiac disease group and healthy controls. Specifically, patients with celiac disease showed an abundance of *Streptococcus, Lactobacillus, Veillonella*, and *Allisonella* species, while the abundance of *Ruminococcus, Faecalibacterium, Blautia, Gemmiger*, and *Anaerostipes* decreased. A comprehensive analysis identified 222 distinct fecal metabolites between the two groups. Using a random forest model, the authors detected four bacterial genera (*Clostridium_IV, Veillonella, Ruminococcus*, and *Gemmiger*) and six metabolites, namely acetylcholine, D-alanyl-D-serine, coronopilin, formiminoglutamic acid, N(6)-methyllysine, and lyso PA (16_0_0_0), as potential biomarkers for the non-invasive diagnosis of celiac disease. A larger sample size will be important for validation purposes.

Li et al. aimed to study the causal relationship between the gut microbiota, its metabolites, and celiac disease using Mendelian randomization analysis. To conduct the study, genome-wide association study summary-level data from previously published studies were examined in a large population (*N* = 18,340) including individuals from diverse ethnic backgrounds. The results of the two-sample Mendelian randomization analysis identified potential causal associations between specific bacterial taxa and metabolites and celiac disease. Increased numbers of *Bifidobacteria* appeared to be linked to a higher risk of celiac disease, while *Lentisphaerae, Coprobacter*, and *Subdoligranulum* showed a potential association with a lower celiac disease risk. The authors provide a correlation between the high or low abundance of these microorganisms in patients with other diseases (IBD, food allergies, autoimmune hepatitis, etc.) and different outcomes on disease onset and progression and suggest that defining the bacterial taxa at a more specialized level (species or strains) could be important. Genetically predicted higher concentrations of five metabolites, namely 1-oleoylglycerophosphoethanolamine, 1-palmitoylglycerophosphoethanolamine, 1,6-anhydroglucose, phenylacetylglutamine, and tryptophan betaine, were strongly linked to higher celiac disease risk, whereas 10-undecenoate and tyrosine were associated with a lower celiac disease risk.

The outcomes of these studies open opportunities for more targeted diagnostics and interventions for disease treatment and provide a basis for further understanding of the underlying mechanisms involved in the disease's pathogenesis.

Liu et al. investigated the impact of the microbial-derived short-chain fatty acid, butyrate, on the regulation of gut mucosal homeostasis using weaned piglets. Dietary supplementation of butyrate resulted in greater diversity and abundance of potential probiotic strains such as *Lactobacillus* and *Blautia* as well as increased gut mucosal barrier function, as shown by an increased relative mRNA expression of intestinal mucosal barrier function-related genes, such as *CLDN1, MUC1, PKC*, and *ITGB1*.

Zhou et al. studied the impact of microbial-derived indole derivatives on the mice model of acute liver failure progression. The authors demonstrated that treatment of mice with acute liver failure with indole-3-acetic acid, indole-3-lactic acid, and indole-3-propionic acid (all microbial metabolites) exacerbated disease progression. In particular, indole-3-acetic acid significantly amplified the inflammatory response and cellular damage via the Tlr2/NF-κB pathway and caused ileal dysbiosis.

Gautier et al. previously showed that *Bacteroides fragilis* or its cell-free supernatant decreased *Salmonella* Heidelberg translocation *in vitro*. In this study, the authors studied the effect of the bioactive fractions of the supernatant on the S. Heidelberg translocation *in vitro* (a model mimicking the intestinal epithelium) and *in vivo* (BALB/c mice). In both models, two fractions strongly decreased S. Heidelberg translocation. In mice, for example, S. Heidelberg was significantly decreased in the spleen and Peyer's patches, which was associated with reduced levels of inflammatory cytokines and neutrophil infiltration. Inhibition of S. Heidelberg translocation also promoted the expression of the tight junction genes in the colon and decreased the numbers of *Alistipes* genus bacteria (which can explain the anti-inflammatory effects of the bioactive fractions as indicated by the IL1-β decrease). The bioactive compounds in fractions (cholic acid and deoxycholic acid) were characterized and quantified through LC-MS/MS and molecular networking analysis. It was shown that cholic acid, in particular, inhibited the translocation of S. Heidelberg by downregulating the *Salmonella* virulence genes *sipA* and *fliC* (flagellar gene).

The study of He et al. focused on the effects of a traditional Chinese medicine, the Shen FuHuang formula, on mice in an acute state of sepsis. The results demonstrated that Shen FuHuang formula treatment increased the survival rate of the mice and hindered the release of pro-inflammatory cytokines (TNF-α, IL-6, and IL-1β). Shen FuHuang formula treatment also improved gut microbiota dysbiosis (decreased the proportion of *Campylobacterota* and *Proteobacteria* but increased *Blautia*). To further investigate the underlying mechanisms, the authors used serum untargeted metabolomics analysis, which suggested that the Shen FuHuang formula could modulate the glucagon and PPAR signaling pathways as well as galactose and pyrimidine metabolism. These results reflect the fact that sepsis, like many other diseases, is mediated by multiple pathways that need to be therapeutically targeted to obtain a therapeutic effect.

Gohil et al. aimed to study bovine-vaginal probiotics genotypically and phenotypically *in silico* and evaluate their performance in 92 buffaloes suffering from clinical endometritis *in vivo*. Two unique vaginal strains, namely *Lactiplantibacillus plantarum* KUGBRC (LPKUGBRC) and *Pediococcus pentosaceus* GBRCKU (PPGBRCKU), were identified and suggested to treat endometritis due to their antimicrobial activity and safety.

In conclusion, the original articles published on this Research Topic highlight the importance and contribution of the gut microbiome in disease pathogenesis. However, these studies were mostly observational and correlative, using preclinical models, emphasizing the necessity of conducting randomized human clinical trials. Given that there is variability in what is considered a “normal” gut microbiome and that the ratios of bacteria that are altered in chronic inflammatory diseases (referred to as dysbiosis) are also not consistent among different studies, this suggests that the molecules that mediate disease progression and resolution probably reside in the composition and levels of gut bacterial metabolites. In this context, understanding the differences in bacterial genus and species composition that define normal and dysbiotic states is a first step to eventually identifying what these bacteria make and what their products do in terms of mediating disease progression and attenuation. The metabolic profiles of different bacterial species in the gut are also important in mediating disease pathogenesis and resolution. For example, different gut bacteria metabolize bile acids into pro- or anti-carcinogenic derivatives, so the ratio of different bacterial species and their corresponding metabolic profiles have a profound influence on cellular and immunological homeostasis, which are associated with health and disease. If this is the case, then future microbiome research will not be about adding back probiotic or recombinant bacteria but about the identification and replacement of biologically active metabolites that mediate disease resolution through the re-establishment of cellular homeostasis (i.e., normal cellular functions) and immunological homeostasis (by promoting anti-inflammatory responses) that define health. In this context, cataloging the gut bacteria associated with normal and disease states at particular organ sites, as outlined herein, is the first step in the later identification of functional metabolites that will have therapeutic value.

## Author contributions

EC: Writing—original draft. MF: Writing—review and editing. AA: Writing—review and editing.

